# Terahertz Spectroscopy of Gas Absorption Using the Superconducting Flux-Flow Oscillator as an Active Source and the Superconducting Integrated Receiver

**DOI:** 10.3390/s20247267

**Published:** 2020-12-18

**Authors:** Nickolay V. Kinev, Kirill I. Rudakov, Lyudmila V. Filippenko, Andrey M. Baryshev, Valery P. Koshelets

**Affiliations:** 1 Kotelnikov Institute of Radio Engineering and Electronics of RAS, Mokhovaya 11-7, 125009 Moscow, Russia; k.rudakov@astro.rug.nl (K.I.R.); lyudmila@hitech.cplire.ru (L.V.F.); valery@hitech.cplire.ru (V.P.K.); 2 Kapteyn Astronomical Institute, University of Groningen, 9712 CP Groningen, The Netherlands; andrey@astro.rug.nl

**Keywords:** superconducting integrated circuits, terahertz emission, terahertz detection, Josephson junctions, gas spectroscopy, absorption lines

## Abstract

We report on the first implementation of a terahertz (THz) source based on a Josephson flux-flow oscillator (FFO) that radiates to open space. The excellent performance of this source and its maturity for practical applications has been demonstrated by the spectroscopy of gas absorption. To study the radiated power, we used a bolometric detection method and additionally calibrated the power by means of pumping the superconductor–insulator–superconductor (SIS) junction, integrated on a single chip with the FFO. For calibration, we developed a program using the SIS-detected power calculations in accordance with the Tien and Gordon model. The power emitted to open space is estimated to be from fractions of µW to several µW in the wide region from 0.25 THz up to 0.75 THz for different designs, with a maximum power of 3.3 µW at 0.34 THz. Next, we used a gas cell and a heterodyne superconducting integrated receiver to trace the absorption lines of water and ammonia with a spectral resolution better than 100 kHz. Our experiment for gas absorption is the first demonstration of the applicability of the FFO as an external active source for different tasks, such as THz spectroscopy, near-field THz imaging and microscopy.

## 1. Introduction

Terahertz technologies are of great importance nowadays due to the extremely wide range of applications: medicine and biology, monitoring of the Earth’s atmosphere and processes in manufacturing, space and atmospheric research, security systems and communication technologies, as well as the fundamental research of materials in physics and chemistry [1,2,3]. Techniques such as terahertz (THz) imaging, time domain, and frequency domain spectroscopy, direct and heterodyne receiving in the THz range, are widely used. Most applications are related in any case to techniques of radiation and detection at THz frequencies, so the sources within this range have been intensively developed and compete with one other in relation to their specific characteristics. Certain new types of THz sources have been proposed and researched in detail over the last 10 years, such as quantum cascade lasers [4,5] and stacks of intrinsic Josephson junctions in high-*T_c_* superconductors Bi_2_Sr_2_CaCu_2_O_8+δ_ [6,7,8,9,10,11]. The technology of resonance tunneling diodes has been significantly developed [12,13], reaching a power of ~10 µW at frequencies around 1 THz, and the semiconductor superlattice multipliers are reported to operate at frequencies of up to 8.1 THz [14,15] with an input frequency of about 100–200 GHz and an operating harmonic number of up to 54. A flux-flow oscillator (FFO), based on a long Josephson junction is a well-researched, on-chip, low-*T_c_* superconducting oscillator, proposed a few decades ago [16,17,18,19] and has been successfully implemented by our group as a local oscillator in the superconducting integrated receiver (SIR) of the 500–650 GHz range [20,21,22,23,24,25,26]. A comparative review of widely used types of THz sources is presented in Table 1. One of the most traditional THz source is a backward wave oscillator, it generates very high power, but the devices operating above 500 GHz are not commercially available due to difficult technology. Nowadays, the most common sources below 1 THz are microwave multipliers based on Schottky diodes, since they combine high power and tuning bandwidth, and are commercially available. In recent years, quantum cascade lasers are progressing rapidly and demonstrate well competitive performance; their operating frequency is roughly within the range of ~1.5 to 6 THz. Photomixers operating at a difference frequency of two lasers are an interesting solution for extremely wideband tuning: they are tuned continuously from 0 to 2 THz, but the power is quite low and decreases exponentially with frequency increase, e.g., from about 50 µW at 200 GHz to about 0.1 µW at 2 THz. Presented in Table 1 are typical numbers not reflecting some special or unique cases.

The FFO operation is based on the ac Josephson effect in an Nb-based long superconductor–insulator–superconductor (SIS) junction having a length *l* which is much greater than the Josephson penetration depth *λ_J_*, accommodating a large number of Josephson fluxes (magnetic vortices) inside the junction equaling approximately *l*/2*λ_J_*, and typically of the order of several tens and larger. One idea relating to the SIR is the on-chip integrating of the SIS-mixer and the widely tunable FFO operating with a phase locking loop and acting as the local oscillator in the THz and sub-THz range [20,21,23]. Recently we proposed and elaborated the idea of utilizing the FFO as an external source of emitting the THz radiation to open space [27,28,29,30] by coupling the junction with an on-chip transmitting lens antenna. As we ensured that the THz emission is provided to open space, the harmonic mixer (HM) for frequency and phase locking were additionally embedded in the integrated structure, resulting in an “FFO and antenna and HM” on-chip structure [27,30]. The main frequency of radiation, *f*, is defined by the Josephson equation

*hf* = 2*eV_DC_*, (1)

where *h* is the Planck constant, *e* is the electron charge, and *V_DC_* is the dc voltage of the Josephson junction. The frequency *f* is widely tunable within the range of 0.25–0.75 THz for the FFO made of Nb/AlO_x_/Nb or Nb/AlN/NbN trilayers. In addition, there are also harmonics that follow the more generalized form of the Josephson equation

ℏ *∂φ*/*∂t* = 2*eV_DC_* (2)

and can be observed indirectly; here, *φ* is the phase difference between the electrodes, known as the Josephson phase and *t* is time. The emission spectral line of the FFO in a free-running regime has a Lorentzian shape with a typical linewidth from a fraction of MHz to several MHz, depending on the operating mode—either a resonant mode with clear Fiske steps on IV-curves or the true flux-flow mode [31,32,33,34].

Both bolometric and heterodyne methods were used to study the output emission to open space. A cooled 4.2K silicon bolometer was used to study the antenna characteristics in the wide band [28], and the SIR with a spectral resolution better than 0.1 MHz was used for precise measurements of the spectral lines at frequencies between 480 GHz and 730 GHz defined by the SIR operating range [27]. Nevertheless, the calibrated emission power was still not measured since the infrared bolometer, used for detection, was not calibrated for the THz range, and there were still some issues related to beam patterns, and hence the “emitter-to-detector” coupling efficiency. It is of interest to study the absolute value of the FFO power emitted to open space. To date, similar research has only been carried out for “on-chip power”, for example, in [35] for the FFO with both NbN-based electrodes, where the absolute power was estimated by detecting the pumping current of the on-chip SIS junction and utilizing the Tien and Gordon model of photon-assistant tunneling in relation to the pumping of the SIS junction by an external THz signal [36,37]. A detected power of 1.3 µW at 760 GHz was obtained in [35]. In this paper, we use an extended technique to evaluate the absolute emitted “power to open space”, using the pumping of the on-chip SIS-based harmonic mixer and known division power ratio between the HM and open space.

Finally, since the FFO has still not been used as an external THz source for applications, we demonstrate its applicability for gas spectroscopy in laboratory conditions with controlled gas pressure. The “active” measurements technique was principally discussed with other type of the THz source, for example, [38,39,40], in which the THz signal is absorbed by the gas and then detected on backend by the receiver, with high spectral resolution. We use the well explored strong absorption lines of water (556.9 GHz) at a pressure of 0.005–20 mbar and of ammonia (572.5 GHz) at a pressure of 0.05–20 mbar in the experiment for gas spectroscopy, utilizing the FFO as an active source. Perfect operational efficiency for spectroscopy, demonstrated in Section 3.2, also opens up possibilities for implementation of the FFO-based source in modern and promising applications, such as near-field THz imaging [41,42,43] and scanning THz microscopy [44,45], for both material research and bio-medical analysis of living tissues.

## 2. Materials and Methods

### 2.1. Device Design Description

**Figure 1:**
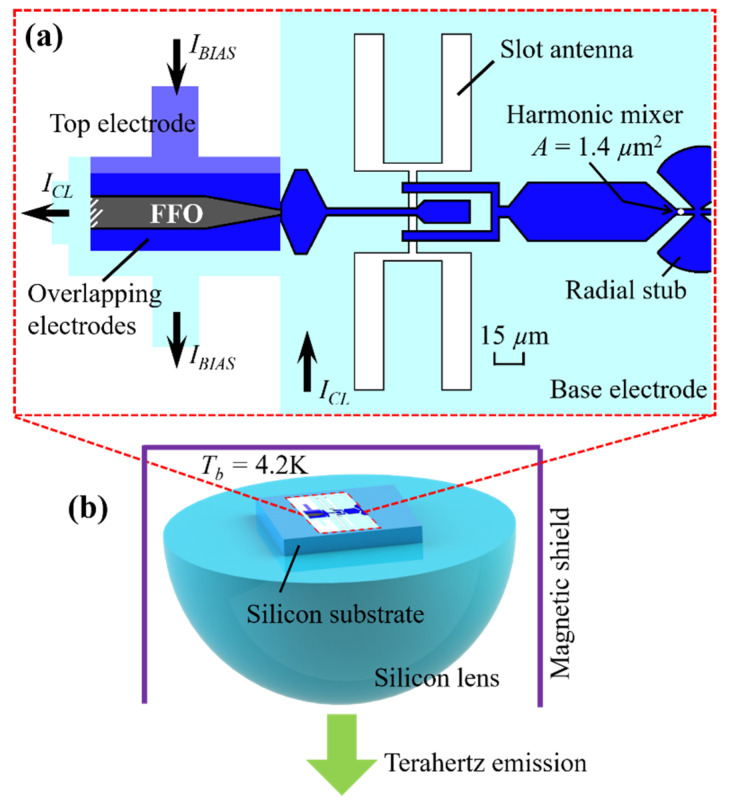
(**a**) Layout of the Nb/AlO_x_/Nb-based integrated microcircuit containing the FFO, the double slot antenna, the harmonic mixer (HM) and the Nb/SiO_2_/Nb-based transmission lines; (**b**) Sketch of emitter microcircuit mounted on a hemispherical silicon lens, transferring the THz emission to open space.

The layout of the “FFO and antenna and HM” integrated structure is shown in Figure 1a, and the schematic of the core of the emitter cryogenic system with the lens, is shown in Figure 1b. The FFO and the HM are fabricated of Nb/AlO_x_/Nb superconducting tunnel structures, with a current density *j_c_* of ~6.5 kA/cm^2^, which corresponds to a normal-state resistance-area product *R_n_* × *A* of ~32 Ω·µm^2^. The dimensions of the FFO are 400 µm × 16 µm with a narrowing at the edges from 16 µm down to ~1 µm for better impedance matching to the output transmission line; the thickness of the AlO_x_ insulation layer is ~1 to 1.5 nm, and the area of the HM is ~1.4 µm^2^. The transmission line is made of Nb/SiO_2_/Nb with a thickness of dielectric SiO_2_ of 400 nm. The base and the top electrodes of both the SIS junctions and the transmission line made of Nb, are fabricated in a single technological process using a magnetron sputtering. Therefore, the FFO and the HM have the same base electrode, and one can see the dc break in the top electrode, close to the center of the slot antenna; hence, the FFO and the HM are controlled by dc independently. The double slot antenna is made in the niobium base electrode; the length and the width of the slots are 182 µm and 15 µm, correspondingly, and the distance between the centers of the slots is 45 µm. The integrated circuits are fabricated on silicon substrate with a dielectric constant of about 11.7. The specific layout presented in Figure 1a is designed for a frequency range of 400–700 GHz; we also developed the designs for the lower ranges of 320–550 GHz [27,30] and of 250–450 GHz [30]. The technology for the fabrication of high quality Nb-based superconducting circuits in our group is discussed in detail in [46,47,48]; this well-developed technology itself is not an area of interest in this paper.

To tune the operating frequency by setting the *V_DC_* according to Equation (1), the FFO is biased by two currents shown schematically in Figure 1a: the current *I_BIAS_* across the tunnel barrier for dc biasing, and the control line current *I_CL_* in the base electrode, required to supply the local magnetic field. To provide the narrow output beam, the chip is mounted on the flat surface of the semispherical 10 mm lens, which together with the substrate, is made of silicon to minimize the reflection and refraction at the chip-lens interface. As the FFO is sensitive to the external magnetic field, the chip is installed inside the superconducting magnetic shield. The cryogenic module presented in Figure 1b is then mounted in liquid helium cryostat, with an operating temperature of *T_b_* = 4.2 K.

### 2.2. Experimental Setup for Studying the THz Emission to Open Space

**Figure 2:**
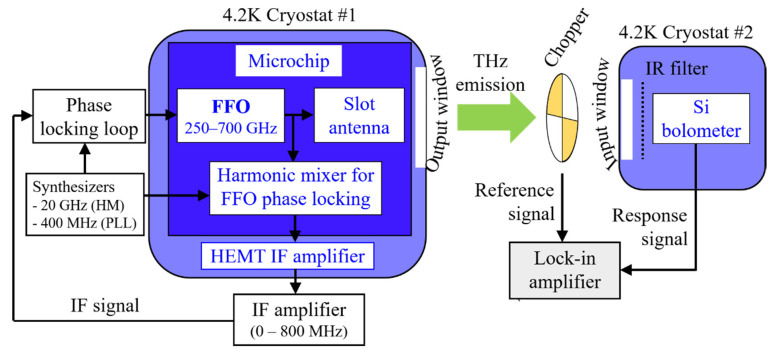
Block diagram of the setup for detecting the emission of the FFO-based source within a range of 250–700 GHz using a cooled Si bolometer. The chopper frequency is around 170 Hz.

The experimental setup for studying the THz emission to open space is presented in Figure 2. Two 4.2 K cryostats are used simultaneously: the left section of the scheme is the FFO-based THz emitter, and the right is the detector. The FFO emission is divided into two fractions by means of microstrip transmission lines: the main fraction (up to 80%) is directed to the lens antenna and then to open space, and certain smaller fraction (about 20%) is branched out to the HM for frequency and/or phase locking. For direct detection, the cooled silicon bolometer is used. As the bolometer is highly sensitive to infrared (IR) radiation, the IR filter on the bolometer input is used to minimize the background signal. The traditional lock-in amplifier technique with an optical chopper modulation, is used to record the bolometer response.

The experiment is carried out as follows: the FFO frequency, *f*, is swept in the wide range by the sweeping of *I_BIAS_* and *I_CL_* currents providing the voltage *V_DC_* to be set within the range of 0.41 mV and 1.65 mV, corresponding to *f* between 200 GHz and 800 GHz (the Josephson constant of about 483.6 GHz/mV can be used for a simple linear conversion between *f* and *V_DC_*). Two experimental values are measured during the frequency sweeping: the bolometer response (not calibrated for the THz region), and the pumping current *I_pump_* of the voltage-biased HM, caused by quasiparticle tunneling in the SIS junction. The HM bias voltage for measurements of *I_pump_* is traditionally set at 2.5 mV, which is close to the gap voltage *V_g_* of ~2.8 mV for Nb/AlO_x_/Nb trilayers and is most suitable for recording the *I_pump_* vs. *f* dependence. Additionally, the HM IV-curves at certain specific FFO frequencies are measured to find the allocated THz power, which will be described in detail in Section 3.1.

### 2.3. Calibration of the Emission Power

There are different approaches for defining the high-frequency power detected by the tunneling SIS junction, some of which are discussed in [37]. At present, the most adequate approach is the photon-assisted tunneling model of Tien and Gordon, discussed in detail, for example, in [36,37]. According to the model, the pumping current of the SIS junction under the influence of the external emission can be defined as the function of the SIS dc voltage *V_DC_* and the frequency of the external signal *f* as

(3) $$I_{pump}(V_{DC},f)=\sum_{n=-\infty }^{+\infty }J_{n}^{2}(\frac{eV_{f}}{hf})I_{DC}(V_{DC}+n\frac{hf}{e})$$

where *J_n_* is the Bessel function of order *n*, *I_DC_*(*V*) represents the autonomous IV-characteristic (with no influence from the external signal) and the *V_f_* is the magnitude of the high-frequency signal across the junction in addition to dc bias:

(4) $$V(t)=V_{0}+V_{f}\cos\omega t$$

If we introduce the dimensionless “pumping parameter”

*α* = *eV_f_*/*hf*, (5)

the “width” of the quasiparticle step on the IV-curve

*V_qp_* = *hf*/*e*, (6)

and use in calculations only the nearest six steps to the “current jump”, which is sufficient for accuracy, then Equation (3) will be written in a simpler form:

(7) $$I_{pump}(V_{DC},V_{qp})=\sum_{n=-3}^{+3}J_{n}^{2}(\alpha )I_{DC}(V_{DC}+nV_{qp})$$

This expression is successfully used for fitting the simulation curves calculated for different *α* and *f* to experimental HM IV-curves under the influence of the FFO emission, and hence for defining *α* and the magnitude of the THz signal, *V_f_*. For this purpose, a program for numerical simulations was developed in Mathcad^®^ 15 [49].

When *α* is known, the power allocated in the HM can be calculated [41] as follows:

(8) $$P_{HM\_abs}=V_{f}{}^{2}/R_{rf}=\alpha^{2}{\left(hf/e\right)}^{2}/R_{rf}$$

where *R_rf_* is the dynamic resistance of the junction at a frequency of pumping, defined as a function of *V_DC_* and *α* as

(9) $$R_{rf}(V_{DC},\alpha )=\frac{2\;V_{qp}}{I_{pump}(V_{DC}+V_{qp},\alpha )-I_{pump}(V_{DC}-V_{qp},\alpha )}$$

where *I_pump_*(*V_DC_*,*α*) is taken from Equation (7) as the function of *α* with constant *V_qp_* which means the constant *f*. Approach Equation (9) is correct for the case of compensated junction capacitance as previously discussed, and an embedded impedance of the external circuits is not taken into account. At certain frequencies, the influence of the embedded impedance can lead to very high or even negative dynamic resistance at the first photon step. In fact, *R_f_* calculated from Equation (9) is slightly lower than the normal resistance *R_n_* of the junction at the voltages of operation and frequencies of pumping, and *R_rf_*/*R_n_* is about 0.8–0.9, with a typical *R_n_* of about 25 Ω for the HM on experimental samples. Finally, the power emitted to open space *P_air_* is calibrated using the absolute power *P_HM_abs_* from Equation (8) at a certain specific frequency, and the known *P_air_*/*P_HM_abs_* ratio taken from numerical results for the sample designs. When the *P_air_* in absolute units is known at some frequency (e.g., 128 nW at 505 GHz, which is certain result for one of the samples and is discussed in Section 3.1), the total frequency dependence measured as a bolometer response in arbitrary units, is calibrated to absolute units. These calculations were made using both Mathcad^®^ 15 for *P_HM_abs_* estimations and OriginPro^®^ 9 [50] for calibration of the bolometer response from arbitrary units to µW.

### 2.4. Experimental Setup for Gas Spectroscopy

**Figure 3:**
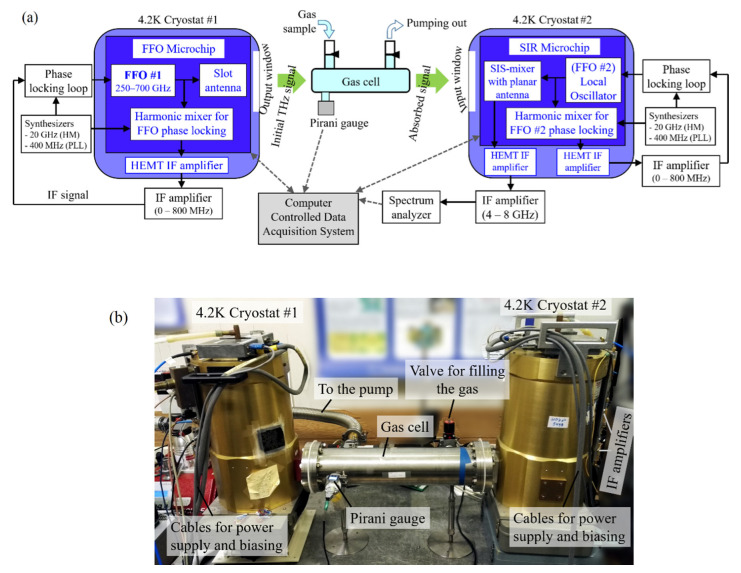
(**a**) Block diagram of the setup for detection of the gas absorption lines by means of the FFO-based active source and the superconducting integrated receiver (SIR)-based THz spectrometer; (**b**) Photo of the setup showing the main parts. Auxiliary parts (computer, synthesizers, biasing and power supply devices, spectrum analyzer etc.) are beyond the photo or on the back of the cryostats.

We carried out the experiment for detection of ammonia and water absorption lines, using the FFO as the active source and the superconducting integrated receiver (SIR) as the THz spectrometer with a high frequency resolution. The complex system for gas detection is shown in Figure 3. A similar tracing technique was used, for example, in [38] with a backward wave oscillator as a THz source, and in [39,40] with a high-*T_c_* superconductor-based source. The output signal from the FFO #1, referred to as the “initial THz signal” passes through the gas and is detected by the backend THz spectrometer after some absorption, caused by rotational transitions. The length of the gas cell is 500 mm, and the cell windows are made of Teflon, transparent in the THz range. The absorption linewidth is highly dependent on the gas pressure that is set by both the filling and pumping systems, and measured by the Pirani gauge. Typical pressure in the experiment is from 10^−3^ mbar to 20 mbar, at higher pressures the absorption can still be detected but the absorption linewidth is considerably larger due to collisional broadening. We should note that there is some additional absorption unrelated to the gas under study, due to the humidity in the room, which is around 35%. The intermediate frequency (IR) range on the SIR is 4–8 GHz with the center at 6 GHz. The spectra are finally recorded by the spectrum analyzer in the IF range.

## 3. Results and Discussion

### 3.1. Emission Power

**Figure 4:**
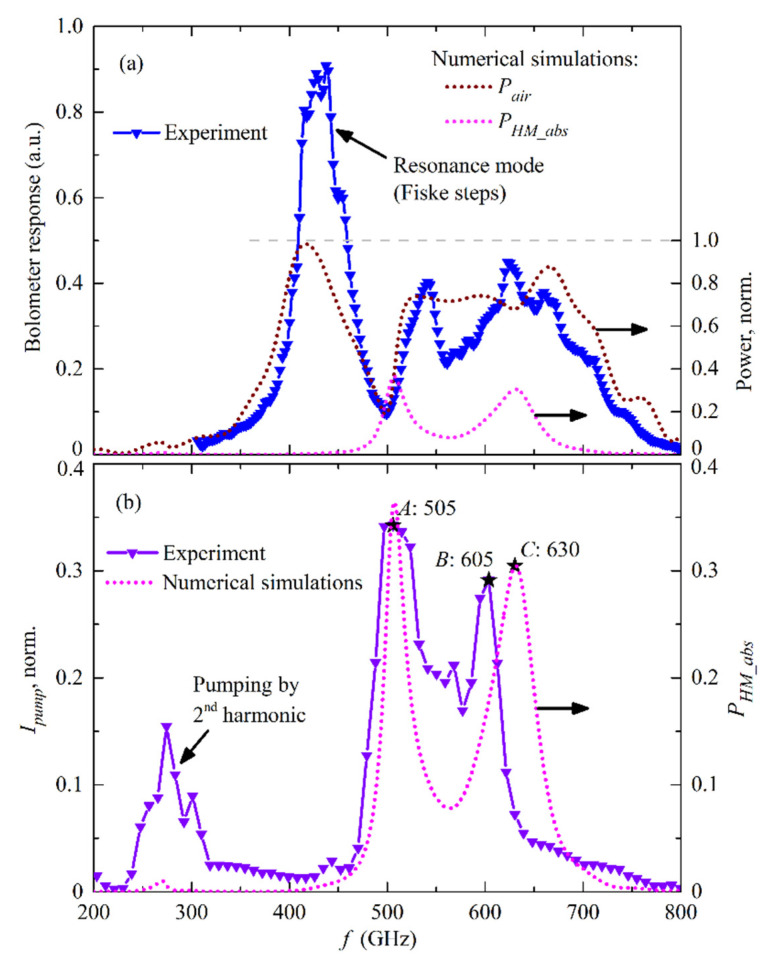
Experimental results compared to numerical simulations of the emitter, designed for the frequency range of 400 to 700 GHz. (**a**) Bolometer response and *P_air_* vs. frequency; *P_HM_abs_* is plotted in addition for clarity; (**b**) HM pumping current *I_pump_* and *P_HM_abs_* vs. frequency. *I_pump_* is normalized to the SIS “current jump” at *V_g_* equal to 144 µA. The curve *P_HM_abs_* is the same in (**a**,**b**) and presented with different scale.

The primary experimental results obtained for the 400–700 GHz design and described in Section 2.2, are presented in Figure 4: the bolometer response is shown in Figure 4a and the HM pumping current in Figure 4b. The results of the numerical simulations are also presented for comparison on the same graphs; the technique used in these high-frequency simulations was discussed in detail in [27,28,29]. Note that the power in numerical simulations is normalized to the full output FFO power at the edge of the long Josephson junction, therefore, the power emitted to open space *P_air_* and absorbed by the HM *P_HM_abs_* cannot be larger than one. Moreover, the sum *P_air_* + *P_HM_abs_* at each frequency also cannot be larger than one, which is marked with a dashed line at the level of one on the right Y-axis in Figure 4a. Herewith, the bolometer response in Figure 4a is presented in a.u. since the detected signal has not yet been calibrated, and the HM pumping current in Figure 4b is normalized to the “current jump” (a sharp current increase at the SIS gap voltage), which is specific for each experimental sample. One can note that both comparisons demonstrate a satisfying correlation between experimental and numerical results, however, there are certain points to discuss. It can be seen that the first experimental peak in the bolometer response at ~420 GHz is about twice higher than the second plateau-like peak with its center at ~600 GHz, which is different from numerical results. This is easily explained by the resonant mode of the FFO at frequencies below *V_g_*/3 [31,32,33,34] with a higher output power than in flux-flow mode at frequencies higher than *V_g_*/3, while in numerical simulations the total FFO power is regarded as being independent of frequency. The “boundary” frequency *f_b_* separating resonant and flux-flow modes for Nb/AlO_x_/Nb-based FFO with *V_g_* = 2.8 mV is *f_b_* = 2*e*/*h*·*V_g_*/3 = 450 GHz, therefore, the peak difference is not an issue. In addition, a certain peak is evident for *I_pump_* (Figure 4b) at low frequencies ~230 to 320 GHz, which is absent in the case of numerical *P_HM_abs_*. This difference is also easily explained by the pumping of the HM by the second harmonic of the FFO. The transmission lines are designed for a frequency range of 400 to 700 GHz, hence, if the main FFO frequency, is for example, *f* = 250 GHz, the second harmonic at *f_2ND_* = 500 GHz is transmitted through the lines and causes the HM pumping current.

Such an agreement between the experimental data and numerical simulations enables an estimation of absolute power to be made, using the known ratio between *P_air_* and *P_HM_abs_* taken from the numerical simulations (dotted curves in Figure 4a). Hence, if we know that the FFO power allocated in the HM at a certain specific frequency at the curve *P_HM_abs_*, we can completely calibrate the power for the entire curves *P_HM_abs_* and the experimental bolometer response, assuming the linear dependence between response and detected power for the bolometer being far from saturation, according to the bolometer specification. Certain specific points *A*, *B* (on the experimental curve) and *C* (on the numerical curve) are marked in Figure 4b at frequencies of 505 GHz, 605 GHz, and 630 GHz, respectively, as reference point for further use.

**Figure 5:**
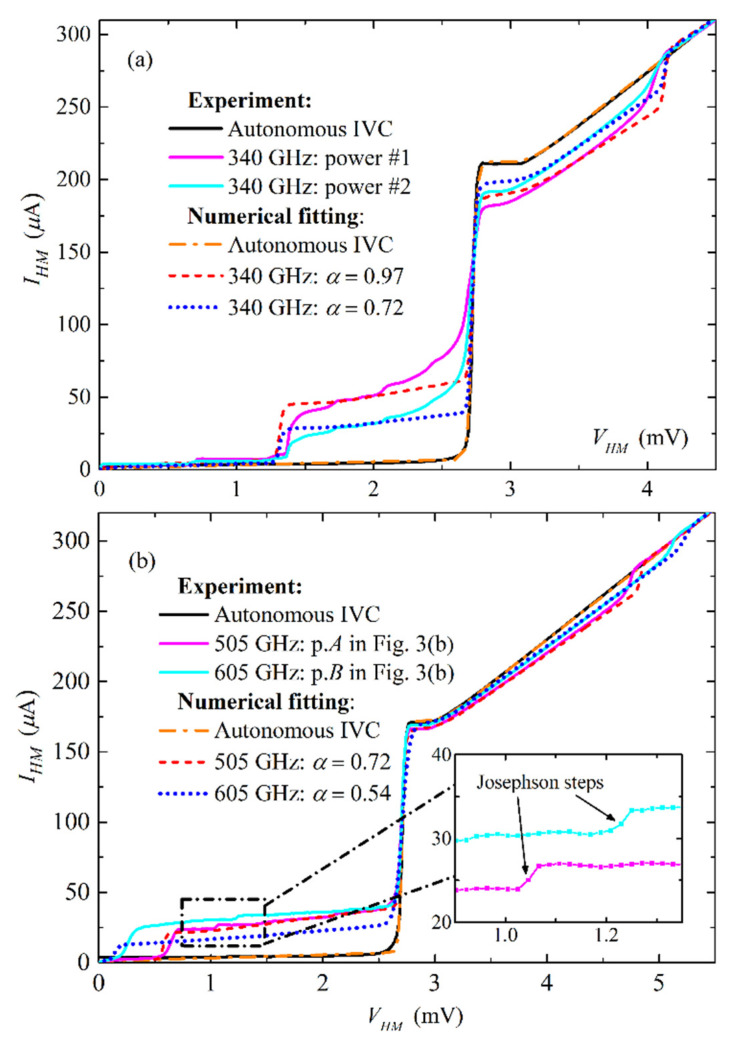
Autonomous and pumped HM IV-curves, experimental (solid) and numerical (dashed and dotted) curves for different experimental samples: (**a**) Sample designed for 250–450 GHz, two pumping powers at 340 GHz are presented from the experiment and simulated with *α* = 0.97 and *α* = 0.72; (**b**) Sample designed for 400–700 GHz, two pumping powers at 505 GHz and 605 GHz are presented from the experiment and simulated with *α* = 0.72 and *α* = 0.54, respectively.

The results for defining of “pumping parameter” α calculated in Mathcad^®^ program using Equation (7) of two different samples are presented in Figure 5. It was observed that the slope of the first steps below the gap with *n* = −1 in experiment and simulations could be different in some cases (Figure 5a) or correlating well (Figure 5b). It should be mentioned that the capacitance of the junction is not taken into account in expressions Equation (3) and Equation (7) since it is usually compensated at operating frequencies by a small inductance of the short microstrip line that is grounded at high frequencies by a radial stub (see Figure 1a). One can also note that a good fit of the step with *n* = +1 does not necessarily lead to a good one for of the step *n* = −1, as presented for pumping at 605 GHz in Figure 5b. Once again, this is due to the simplified models Equation (3), Equation (7) and also due the critical (superconducting) current of the junction not being completely suppressed, which leads to additional, small, clear Josephson current steps at ~1 mV for 505 GHz and ~1.2 mV for 605 GHz pumping (see inset in Figure 5b). The experimental curves in Figure 5b correspond to points *A* and *B* in Figure 4b.

**Figure 6:**
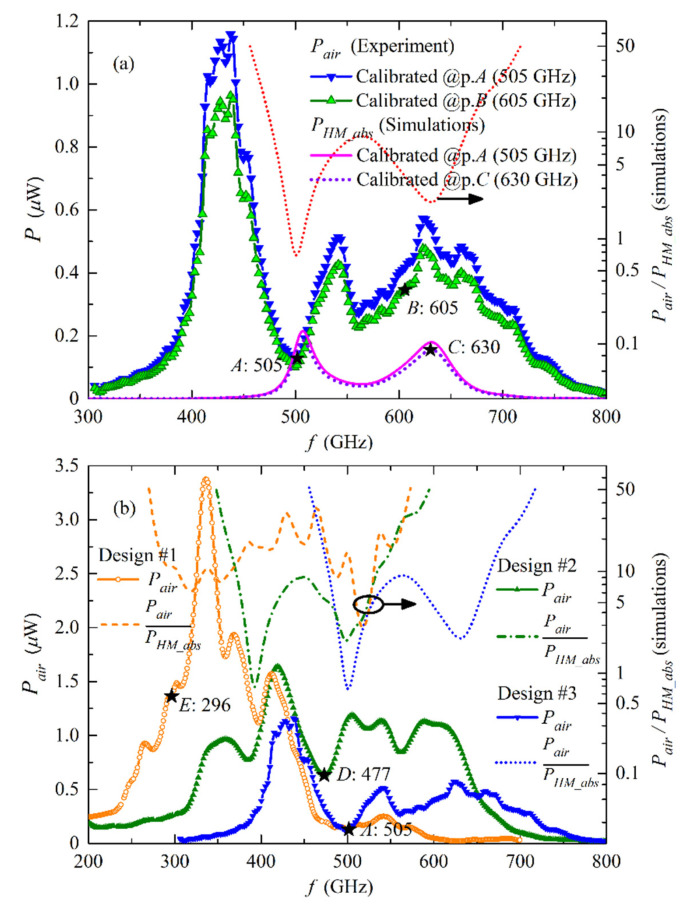
Calibrated power to open space: (**a**) For experimental sample designed for 400–700 GHz, calibration at points *A* and *B* is shown, calibration for HM absorption power at point *C* is also presented; (**b**) For samples of three different designs, the points *D* and *E* where calibration was made, are marked.

The results for calibration of the power emitted to open space using the Equation (8) for *P_HM_abs_* and the known ratio *P_air_*/*P_HM_abs_*, are presented in Figure 6. As expected, the power calibrated at different points of HM pumping is slightly different (see blue and green curves in Figure 6a, calibrated at points *A* and *B*, respectively). Nevertheless, this difference is commonly not higher than 50% for all obtained experimental data, so this accuracy is sufficient for estimation. In Figure 6b the results for different emitter designs are presented: design #1 for 320–550 GHz (orange curve), design #2 for 250–450 GHz (green curve) and design #3 (blue curve) for 400–700 GHz repeated from Figure 6a. For all designs #1–3 the power emission at frequencies below 450 GHz (*V_g_*/3) is noticeably higher than that for frequencies above *V_g_*/3, which is clearly explained by the Fiske resonances [31,32,33,34]. The results obtained in this section allow us to state that the output emission power of the developed FFO-based THz source ranges from fractions of µW up to several µW, these results are in agreement with [35]. A maximum power of 3.3 µW at 0.34 THz emitted to open space and detected by the bolometer is obtained on the sample of design #3 for lower frequency range.

### 3.2. Absorption Lines of Water and Ammonia

**Figure 7:**
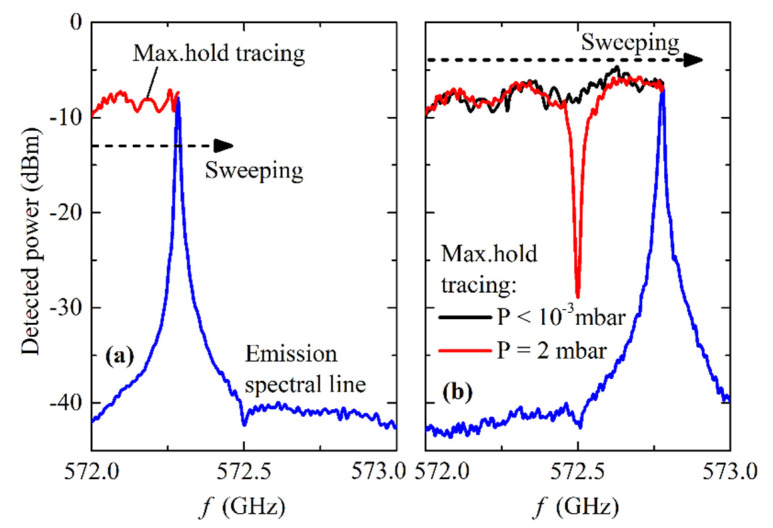
Recording of the gas absorption lines by the “maximum hold” regime of the spectrum analyzer, using a frequency sweeping of the FFO emission line: (**a**) The emission line still did not pass the frequency of absorption; (**b**) The emission line passed the frequency of absorption.

The recording of absorption lines is carried out as follows: the frequency of the SIR local oscillator (FFO #2 in Figure 3a) is set at 6 GHz higher or lower than the absorption frequency, e.g., at 578.5 GHz for ammonia detection at a frequency of 572.5GHz, and the frequency of the active source (FFO #1 in Figure 3a) is slowly and continuously swept within a low range of ±0.5–1 GHz around the absorption frequency, e.g., from 572.0 GHz to 573.0 GHz and back again. The “maximum hold” tracing mode is switched on in the spectrum analyzer (model Agilent/Keysight™ E4440A) while sweeping the FFO frequency, so the emission peak is recorded at each frequency in the sweeping range, as demonstrated in Figure 7. If the gas is filled in the cell and the pressure is sufficient for detection, the clear absorption minimum is recorded as the red curve, shown in Figure 7b. If no absorption is detected, a relatively flat reference curve is recorded (drawn in black in Figure 7b) which is used as a “zero”-curve for further processing. Since the IF background vs frequency is not absolutely flat due to the characteristics of the IF amplifiers and standing waves in the IF circuit of the SIR, the “maximum hold” trace of the emission peak is also not completely flat. We repeat this procedure at different pressure levels of the gas, and then numerically subtract the “zero”-curve corresponding to no absorption from the recorded curve at a certain level of pressure; this leads to pure absorption characteristics which are counted relative to 0 dB.

**Figure 8:**
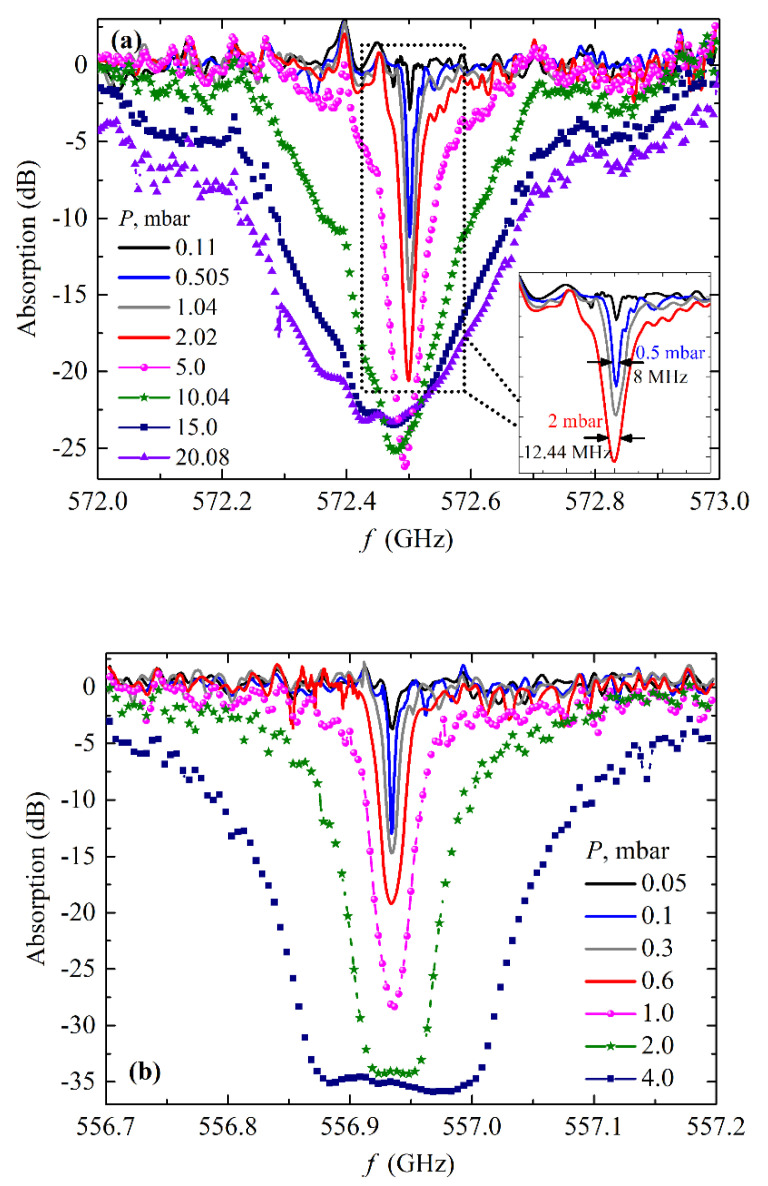
Absorption spectra at different pressures of ammonia, 10% water solution, (**a**) and distilled water (**b**), detected by the FFO- and the SIR-based tracing system.

The results are presented in Figure 8a for ammonia (NH_3_) and in Figure 8b for water (H_2_O) vapor. As for the sample of NH_3_, we used the available water solution at a proportion of 10%, hence the partial pressure of NH_3_ in the vapor mix was less than that measured by the Pirani gauge and specified in the legend (Figure 8a). In the case of distilled water, the measured pressure is the true pressure of the H_2_O vapor. It was observed that the absorption lines were broadened and enhanced with an increase in pressure; an absorption linewidth defined at a half power of the peak (or, the same, at the level of −3dB/+3dB for positive/negative Y-axis peak direction) for certain pressures is shown by the inset in Figure 8a, and is equal to ~8 MHz and ~12.5 MHz at 0.5 mbar and 2 mbar, respectively. Using this technique, we detected NH_3_ at the level of vapor pressure as low as 0.05 mbar, and for H_2_O as low as the vapor pressure of 0.005 mbar, which could be sufficient for practical application. The results are in good agreement with [38]. The observed rotational-transition frequencies coincide with the values from existing literature and databases on molecular absorption [51,52]. We present the supplementary materials Video S1 alongside this paper, demonstrating the real-time process of gas detection during FFO frequency sweeping, for both NH_3_ and H_2_O vapors (see Video S1).

For discussion, we should note certain points regarding this technique. Firstly, the linewidth of the FFO emission lines does not really matter in the experiment; however, the frequency-sweeping step must be lower than the linewidth for accurate “maximum hold” tracing. The FFO linewidth was around 15 MHz in the experiment with NH_3_ (the blue curve in Figure 7) and around 12 MHz in the experiment with H_2_O. Secondly, we should note that neither the frequency nor the phase locking of the FFO is implemented in this experiment, despite the fact that such locking is realized for the emitter design by using the HM. Actually, a reasonably wider FFO spectral line can lead to more accurate results since it can cover the frequency points more times during the sweeping process. The wide-linewidth sources are used in a noise spectroscopy [40,53,54]. The overall spectral resolution of the method implemented in this paper is determined by the resolution of the SIR with phase locked FFO, which is better than 100 kHz [25]. Thirdly, we should note that our experiment demonstrates a close spectral resolution and sensitivity to a trace gas system, based on quantum-cascade lasers reported in [55]; it has an even better resolution and accuracy of an absolute frequency definition, than a system based on chirped-pulse Fourier-transform spectrometers [56]. Finally, the pressure sensitivity that we demonstrate (0.05 mbar for NH_3_ and 0.005 mbar for H_2_O) can be increased by two or three orders of magnitude using another backend system and/or a modulation of the FFO-based active source. In the present experiment, we used the spectrum analyzer as the backend for simple demonstration of the absorption, so the presence/absence of the gas was estimated only visually on the screen, though this technique is far from being optimal from the sensitivity point of view. Instead of the spectrum analyzer, a power meter or, the better, a digital fast Fourier transform spectrometer should be used to improve the sensitivity. Furthermore, an accumulating of the signal for longer time and larger number of sweeping the frequency can definitely increase the sensitivity. As the source emitting to open space, presented in this paper is widely and relatively easy tunable, and its output beam is focused by the lens antenna and can be designed for a specific task, this source can be used in wide range of other applications besides the gas spectroscopy. The compact cryogenic module with the emitter can be installed in a single setup with the surfaces and materials under investigation at low temperatures, utilizing the near-field scanning microscopy or terahertz imaging techniques [41,42,43,44,45].

## 4. Conclusions

In this work, we carefully studied the THz emission to open space of the flux-flow oscillator, integrated with the lens antenna and presented a system for tracing the gas absorption lines in the THz region, using the FFO-based active source and the SIS-based high-resolution spectrometer. At the first stage, we measured a frequency dependence of the FFO output emission using a cooled silicon bolometer and a frequency dependence of pumping of the harmonic mixer, located on a single chip with the FFO, within a range of between 250 GHz and 750 GHz for three designs. Then we calculated the power absorbed by the HM using the Tien and Gordon model of photon-assistant tunneling, and calibrated the power emitted to open space, which is of the order of a fraction of µW to several µW, depending on the frequency and the operating mode. The maximum power of 3.3 µW at 340 GHz was obtained for the design operating at 320–550 GHz. At the final stage, we developed a system for gas sensing using the spectroscopy of absorption lines, utilizing the developed FFO-based emitter and the superconducting integrated receiver as the THz backend spectrometer. We recorded the clear absorption lines of ammonia and water vapor, at 572.5 GHz and 556.93 GHz, respectively, with a resolution of around 100 kHz, and observed a broadening and enhancing of lines with an increase in pressure. This is the first demonstration of gas spectroscopy under laboratory conditions, utilizing the FFO as an active THz source. Certain new possible applications of the emitter are proposed, such as near-field terahertz imaging and microscopy, which are promising techniques in many fields.

## Figures and Tables

**Table 1:** Typical characteristics of different types of THz sources.

	Type of a Terahertz Source					
Properties	Backward Wave Oscillators	Multipliers Based on Schottky Diodes	Quantum Cascade Lasers	Photo-Mixers	High-*T_c_* Josephson Oscillators	Low-*T_c_* Josephson FFOs
Typical operating frequencies	0.1–1.5 THz	up to ~2 THz	1.5–6 THz	up to 2 THz	~0.5 to 1 THz	250–900 GHz ^1^
Tuning bandwidth related to carrier frequency	up to 30%	~20 to 30%	0.1–10%	100%	up to 50%	up to 80%
Typical power	up to tens of mW	up to several mW	up to 1 mW	tens of nW–several µW	up to tens of µW	tens of nW–several µW
Operating temperatures	~293 K	~293 K or cryogenic	4.2–50 K	~293 K or cryogenic	~20 to 60 K	4.2 K
Main disadvantages	extremely large weight and power consumption	low harmonic efficiency above 1 THz	not available below 1 THz	for high power, pulse source are needed	wide spectral line, phase locking is still not available	extremely sensitive to temperature changes and external field

^1^ Upper limit is ~700 GHz for Nb/Nb-based and ~900 GHz for NbN/NbN-based flux-flow oscillators (FFOs).

## Supplementary Materials

The following are available online at https://www.mdpi.com/1424-8220/20/24/7267/s1, Video S1: The real-time gas tracing process recorded during the FFO frequency sweeping for NH_3_ and H_2_O.
